# A case management model for patients with granulomatous mastitis: a prospective study

**DOI:** 10.1186/s12905-022-01726-w

**Published:** 2022-05-02

**Authors:** Yuan Deng, Ying Xiong, Ping Ning, Xin Wang, Xiao-Rong Han, Guo-Fang Tu, Pei-Yu He

**Affiliations:** 1 Department of Breast, Chengdu Women’s and Children’s Central Hospital, School of Medicine, University of Electronic Science and Technology of China, No. 1617, Riyue Avenue, Qingyang District, Chengdu, 611731 Sichuan Province People’s Republic of China; 2 Department of Nursing, Chengdu Women’s and Children’s Central Hospital, School of Medicine, University of Electronic Science and Technology of China, No. 1617, Riyue Avenue, Qingyang District, Chengdu, 611731 Sichuan Province People’s Republic of China

**Keywords:** Granulomatous mastitis, Case management, Recurrence, Medication adherence

## Abstract

**Background:**

Granulomatous mastitis (GM) is a chronic inflammatory mastitis disease that requires long-term treatment and has a high recurrence rate. Case management has been proven to be an effective mechanism in assisting patients with chronic illness to receive regular and targeted disease monitoring and health care service. The aim of this study was to investigate the application of a hospital-to-community model of case management for granulomatous mastitis and explore the related factors associated with its recurrence.

**Methods:**

This was a prospective study on patients with granulomatous mastitis based on a case management model. Data on demographic, clinical and laboratory information, treatment methods, follow-up time, and recurrence were collected and analyzed. The eight-item Morisky Medication Adherence Scale (MMAS-8) was used to investigate patients' adherence to medications. Logistic regression models were built for analysis of risk factors for the recurrence of granulomatous mastitis.

**Results:**

By October 2021, a total of 152 female patients with a mean age of 32 years had undergone the entire case management process. The mean total course of case management was 24.54 (range 15–45) months. Almost all the patients received medication treatment, except for one pregnant patient who received observation therapy, and approximately 53.9% of the patients received medication and surgery. The overall recurrence rate was 11.2%, and “high” medication adherence (RR = 0.428, 95% CI 0.224–0.867, *P* = 0.015) was significantly associated with a lower rate of recurrence, while the rate of recurrence with a surgical procedure + medication was higher than that with medication alone (RR = 4.128, 95% CI 1.026–16.610, *P* = 0.046).

**Conclusion:**

A case management model for patients with granulomatous mastitis was applied to effectively monitor changes in the disease and to identify factors associated with disease recurrence. “Low” medication adherence was a significant risk factor for the recurrence of granulomatous mastitis. Patients treated with medication and surgery were more likely to experience recurrence than those treated with medication alone. The optimal treatment approach should be planned for granulomatous mastitis patients, and patient medication adherence should be of concern to medical staff.

## Background

Granulomatous mastitis (GM) was first reported as a chronic inflammatory disease of the breast by Kessler and Wolloch in 1972 [1], and accounts for approximately 1.8% of benign breast diseases [2]. The main clinical presentation is a palpable, painful breast lump with concomitant skin erythema, nipple retraction, sinus tract formation, cellulitis changes, and axillary adenopathy formation [3–5], and in severe cases, there are usually multiple coexisting focal abscesses with skin inflammation and ulceration [5]. According to the severity of the disease, GM is clinically classified into mass, abscess, and refractory types [6]. Patients often endure a long disease course, as well as changes in breast appearance caused by the disease, which has serious physical and psychological effects on patients [7]. With only 2.4 per 100,00 incidences reported by the Centers for Disease Control and Prevention in 2009, most countries have not conducted large epidemiological surveys for GM due to the rarity of the disease [8]. To date, the etiology of GM is unknown and may be associated with a history of pregnancy, autoimmune disease, breast trauma, hyperprolactinemia, and infection [7, 9]. The disease progresses rapidly with a recurrent or prolonged natural course, which has a high recurrence rate of 5%-50%, and is commonly seen in young women with a history of breastfeeding and childbirth [3, 10–12]. As recently reported, there are racial differences in this disease, and the incidence of GM in Middle Eastern countries (Egypt, Turkey, Iran) and Spain is higher than that in Western countries (UK, USA, New Zealand) [13–15]. A large number of cases of GM have been described, mainly from Asian and Mediterranean countries, such as China, Iran, and Turkey [16, 17]. However, there is no consensus on the management of GM and no gold standard regarding the diagnosis and treatment of the disease [4]. Currently, the main treatment include observation, medication therapy (steroids, antibiotics, methotrexate (MTX), and anti-molecular bacilli) and/or operative interventions (abscess incision and drainage, simple mass excision, enlarged mammary mass excision, etc.) [15, 18], and medication therapy is the most commonly used treatment. The toxic side effects of long-term medication use have a significant impact on patients' quality of life, resulting in poor compliance with drug use, therefore, timely observation of medication use and changes in the breasts is essential to achieve good recovery rates for GM patients [11, 18–20].

Recently, one approach to managing care that has gained wide popularity is case management [21], which promote access to provide patients with regular and targeted disease monitoring and health guidance through follow-up visits and WeChat consultations in China (WeChat is a mobile chat software by the Chinese company Tencent, in which patients can quickly consult with medical staff by sending voice messages, videos, pictures and texts over the internet quickly) [22]. Nurse specialists are responsible for the overall coordination, management, and continuity of care for a specific treatment or intervention to meet the health needs of an individual, reduce health care costs and improve the quality of service [23, 24]. Currently, it is known that case management is widely applied for patients with breast disease, especially breast cancer [25, 26], but it is rarely to applied for GM patients. Based on the characteristics of the disease, which is mostly treated and followed up in outpatients, a tailored model should be developed that it enables health providers monitor the condition changes of GM patients from outpatient to community to inpatient settings. A hospital-to-community model of case management, which allows cases managers to track and manage the treatment of GM patients from hospital to community settings, was described by Lamb in 1992, and includes the following five basic activities of case management: (1) assessment, (2) planning, (3) linking, (4) monitoring, and (5) advocacy [27]. Since January 2018, a tailored model for GM based on a hospital-to-community model, which can provide patients with full management and seamless health care services, has been explored and practiced in Chengdu Women's and Children's Central Hospital.

### Purpose

To better observe the development of this disease with treatment and identify some of the factors associated with its recurrence, we used a hospital-to-community-based model of case management to monitor the condition changes of GM patients. Prospective studies can provide more effective strategies and optimal approaches to prevent the recurrence of disease.

## Materials and methods

### Study design and participants

**Fig. 1:**
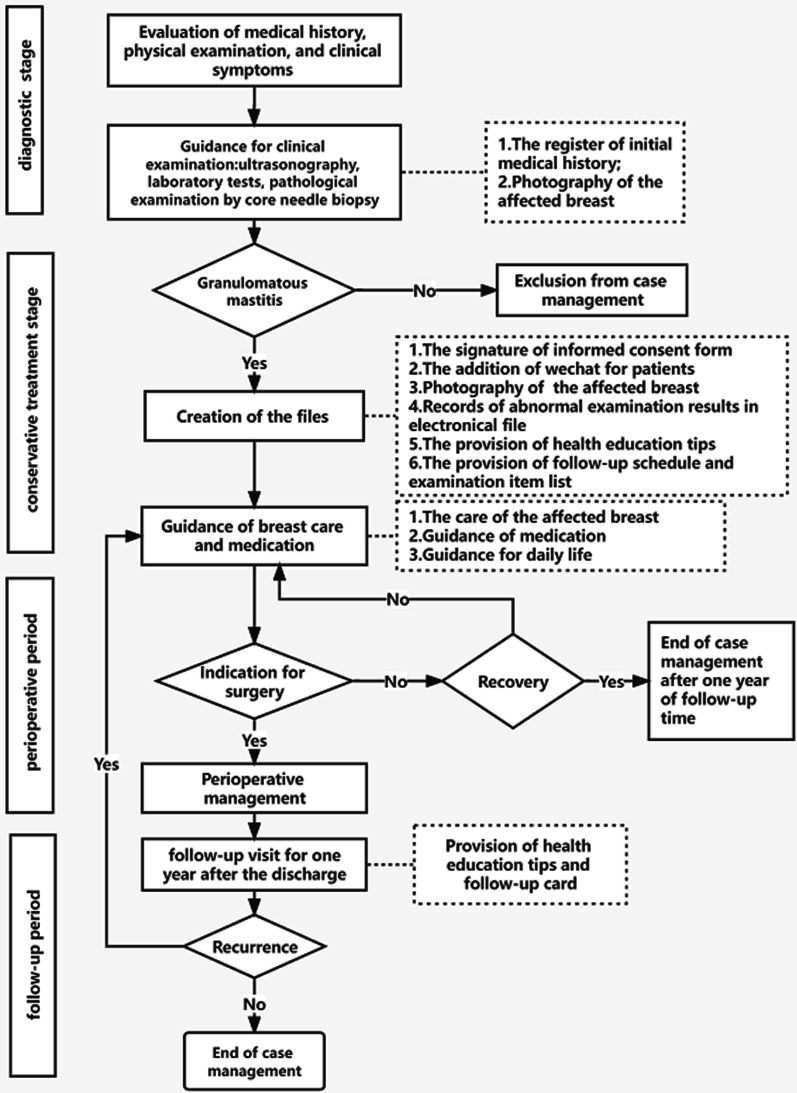
the algorithm for the case management of granulomatous mastitis

A prospective study on patients with granulomatous mastitis based on the case management model was undertaken between January 2018 and November 2020 in the Breast Unit of Chengdu Women’s and Children’s Central Hospital. According to the characteristics of the disease, the whole case management process, presented in Fig. 1, was divided into four key stages, including the diagnostic, conservative, perioperative, and follow-up periods. The entire process was led by case managers and tailored for patients, including the evaluation, planning, integration, implementation, and evaluation of treatment plans. Participants were followed up through the whole process. The case closure time was defined as the time when a patient was free of relapse during the 1-year follow-up period after the discontinuation of medication or surgery.

In the diagnostic stage, the case managers mainly based their decisions on clinical symptoms, regardless of whether a register of the initial medical history was created including age, pregnancy history, disease history, onset time, onset trigger, and contact phone number. A patient’s diagnosis of granulomatous mastitis was confirmed by the results of a pathological examination by core needle biopsy, and then a case management file was established. In the conservative treatment stage, case managers mainly performed the following: (1) followed up and recorded the results of ultrasounds, abnormal laboratory tests and breast signs, and explained the precautions and methods of medication administration according to a doctor's prescription; (2) surveyed GM patients for medication adherence at 2 months of drug use by the eight-item Morisky Medication Adherence Scale (MMAS-8) [28]; (3) distributed notes of disease considerations related to diet, sleep, behaviors, etc., as shown in Table 1 [6, 29, 30]; and (4) established a contact platform for GM patients to understand and observe the changes in their breasts during treatment, while being given psychological support and guidance at home. In the perioperative and follow-up periods, the case managers recorded the patients' surgery, medication, follow-up time and recurrence information.

**Table 1:** Notes of disease considerations

Contents	Cautions
Dietary [14, 35]	Meat: meat other than pork Seafood: seaweed, laver, etc. FaWu (name from China): mushrooms, bamboo shoots, etc. Tropical fruits: cinnamon, mango, durian, etc. Spicy food: hot pot, barbecue, etc.
Behavior [27, 28]	Breast collision Consumption of alcohol and tobacco Poor sleep and emotions Pregnancy during the treatment period

In this study, qualifications for case managers were as follows: (1) nurses with bachelor's degree or above; (2) nurses with an intermediate title or above; (3) nurses with 5 years of experience or more in the breast department; (4) nurses who had received the training, which included the case management process, communication and health promotion skills; (5) nurses who were required to rotate through the breast clinic, ultrasound and pathology department, wound care unit and operating room, and (6) nurses who had passed the hospital examination for case management. All patients who received case management were eligible for inclusion if they were older than 18 years, had clinical breast symptoms, and had a confirmed diagnosis by core needle biopsy. Patients were ineligible if they had other complications of the breast and had been treated at other institutions. The study was approved by the Ethics Committee of Chengdu Women's and Children's Central Hospital (No. B2019 (13)). All participants signed an informed consent form.

### Case definition

Histopathological examination is a necessary and gold-standard method for the diagnosis of granulomatous mastitis [31], so a definitive diagnosis of GM was largely accomplished with core needle biopsy in this study. The disease may be locally invasive with a risk of recurrence, and recurrence rates of 5 to 50% have been observed by various studies in recent years [10–12]. The following definition of recurrence was used in this study: the detection of new lesion (s) within the range of the primary location or any other part of the ipsilateral breast 1 month following the termination of therapy.

Medication adherence was measured using the eight-item Morisky Medication Adherence Scale (MMAS-8) [28], which was translated into a Chinese scale by Lin Chen et al. [32]; this scale has high reliability and validity and has been widely used in studies of various chronic diseases in China [32, 33]. Three levels of adherence were considered based on the following scores: 0 to < 6 (low); 6 to < 8 (medium); and 8 (high). In a meta-analysis by Lei et al. [34], oral drug therapy was an effective treatment modality or GM patients in receiving both surgical and conservative treatment. According to the relevant literature, steroids are the most prominent drugs for GM, which usually lasts from 3 to 12 months, with a minimum of 2 months [35–38]. To survey as many patients as possible, we chose to conduct a survey of medication adherence at 2 months of medication use.

### Statistical analysis

The statistical software package SPSS for Windows, version 19.0 (SPSS Inc., Chicago, IL) was used for statistical analysis. Clinical characteristics were described using the mean ± standard deviation, the mean (range) or numbers (and percentages) as appropriate. Continuous variables were compared between patients with and without recurrence using one-way ANOVA, while categorical variables were compared using the Chi-square test and Fisher's exact tests. Logistic regression models were built for the analysis of risk factors for the recurrence of GM.

## Results

### Patient characteristics

In this study, 204 symptomatic patients with granulomatous mastitis were initially included in the diagnostic stage between January 2018 and November 2020. However, 4 patients were diagnosed with breast cancer, 8 dropped out, and 40 were still undergoing case management. Ultimately, 152 patients had completed case management by September 2021. Table 2 shows that the mean age of the patients was 32 years (range 22–48). It was observed that 71 (46.7%) patients had normal BMI, while 64 (47,4%) patients had a BMI higher than 25, and were considered overweight or obese. It was detected that the period in which GM was most frequently seen was the first 2–5 years after birth, with 94 patients (61.8%), followed by 30 patients (19.7%) diagnosed 0–2 years after birth (4 patients were breastfeeding), and 15 patients (9.9%) diagnosed during pregnancy. Accompanying diseases were found in only 28 (18.5%) patients, such as diabetes mellitus, thyroid disease, psychoses, hypertension, and hyperprolactinemia, accounting for the highest percentage of 13.8% of all comorbidities.

**Table 2:** Demographic and clinical characteristics of 152 patients

Variables	Patients (n = 152)
*Age, mean (SD) [range], years*	32 (4) [22–48]
*BMI (kg/m*^*2*^*)*	
≥ 30	8 (5.3%)
25–30	64 (42.1%)
18.5–24.99	71 (46.7%)
< 18.5	9 (5.9%)
*Time interval between birth and onset of GLM (years)*	
Pregnancy	15 (9.9%)
Breastfeeding	4 (2.6%)
Time until last birth (years) (< 2, no breastfeeding)	26 (17.1%)
Time until last birth (years) (2–5)	94 (61.8%)
Time until last birth (years) (> 5)	8 (5.3%)
No birth	5 (3.3%)
*Comorbid disease*	
Diabetes mellitus	1 (0.7%)
Thyroid disease	1 (0.7%)
Psychoses	3 (2.0%)
Hypertension	2 (1.3%)
Hyperprolactinemia	21 (13.8%)
*Side*	
Left	81 (53.2%)
Right	59 (38.9%)
Bilateral	12 (7.9%)
*Sign and symptoms*	
Palpable mass with pain	150 (98.7%)
Skin lesion	114 (75%)
Abscess	64 (38.8%)
Fistula	8 (5.3%)
Erythema nodosum	8 (5.3%)
*Mass size at onset* [43]	
< 1 cm	6 (4.2%)
1–2 cm	40 (26.3%)
3–5 cm	69 (45.6%)
> 5 cm	36 (23.9%)
*Laboratory tests before treatment*	
*C. kroppenstedtii*	
Positive	36 (23.68%)
White blood cell (WBC) mean [range], *10^9^/L	9.77 ± 3.33 (3.59–17.55)
C-reactive protein (CRP) mean [range], mg/L	11.91 ± 18.91 (1.0–139)
Prolactin (PRL) mean [range], ng/mL	22.70 ± 19.72 (3.5–129.24)
*Types of GM*	
Mass type	74 (48.7%)
Abscess type	66 (43.4%)
Refractory type	12 (7.9%)
*Behavior before the onset*	
Breast trauma	13 (8.6%)
Excitant food	22 (14.47%)
Staying up all night	12 (7.89%)

On physical examination, the most common finding was a palpable mass with pain (98.7%); 38.8% of the patients had a breast abscess, 75% suffered from skin lesions, and approximately 5% had fistulas and erythema nodosum (Table 2). Based on clinical symptoms, the disease was typed as the mass (74, 48.7%), abscess (66, 43.4%), and refractory types (12, 7.9%). Unilateral involvement was observed the most in 140 (92.1%) patients. In this study, 30.96% of the patients reported that they had bad behaviors a week before disease onset, including breast trauma (8.6%), excitant food (14.47%), and staying up all night (7.89%).

### Patient treatments

Table 3 shows the different treatments that were administered. Of the 152 patients, only 1 (0.7%) recovered under observation without treatment, 82 (53.9%) recovered with medication and surgery, and 69 (45.4%) recovered with medical treatment alone. In the courses of medications, 65 (42.8%)patients chose systemic steroids alone, 21 (13.7%) patients chose tubercle bacillus drugs alone, and 65 (42.8%) patients required a combination or change of the drug regimen due to ineffective treatment or drug side effects including erythema nodosum (5.3%), skin rash (5.3%), abnormal index of liver function (7.2%), abnormal uric acid (2.0%) and edema on the lips and face (0.7%).

**Table 3:** Treatments of patients

Variables	Patients (n = 152)
*Treatments*	
Medication + surgery	80 (52.6%)
Medication	71 (46.7%)
Observation	1 (0.7%)
*Medication*	
Single steroids	84 (55.6%)
Single tubercle bacillus drug	20 (13.2%)
Combined medication (Antibiotics and/or steroids and/or methotrexate and/or tubercle bacillus drug and/or immunosuppressants and/or bromocriptine)	47 (31.2%)
*Surgery*	
Breast lesion excision by minimally invasive surgery	20 (13.2%)
Breast lesion excision by open surgery	60 (39.5%)
Without surgery	72 (47.4%)
*Side effects*	
Skin rash	8 (5.3%)
Abnormal index of liver function	11 (7.2%)
Abnormal uric acid	3 (2.0%)
Edema on the lips and face	1 (0.7%)

### Patient follow-up visits

The mean follow-up time was 25.55 months (range 15–45) for the patients treated with medication and surgery, while it was 23.83 months (range 17–36) for the patients treated with medication alone. There was no statistically significant difference between the groups (*p* = 0.570). The recurrence rate in the series was determined to be as 11.2% with 17 patients experiencing recurrence. At 2 months of initial medication use, the medication adherence outcome of the GM patients was “high” for 59 patients (39%), “medium” for 70 patients (46.4%), and “low” for 22 patients (14.6%), as shown in Table 4.

**Table 4:** Follow-up visit of patients

Variables	Patients (n = 152)
*Follow-up time, mean (SD) [range], m*	24.54 (4.38) [15–45]
Medical + Breast lesion excision by minimally invasive surgery	26.38 (8.27) [15–45]
Medical + Breast lesion excision by open surgery	24.72 (3.97) [17–36]
Medical	23.83 (2.62) [16–30]
Observation	12
*Recurrence*	
Yes	17 (11.2%)
No	135 (88.8%)
*Medication adherence*	
High	59 (39.0%)
Median	70 (46.4%)
Low	22 (14.6%)

### Factors associated with recurrence

All statistically significant variables (*P* < 0.05) related to BMI, treatments, medication use and medication adherence (Table 5) were included in the multivariable logistic regression model. The results of the multivariable analysis are shown in Table 6. Surgical procedure and drug treatment (RR = 4.128, 95% CI 1.026–16.610, *P* = 0.046) were independently associated with an increased recurrence risk of granulomatous mastitis. In contrast, “high” medication adherence (RR = 0.428, 95% CI 0.224–0.867, *P* = 0.015) was associated with decreased recurrence risk.

**Table 5:** The characteristics in GM patients with and without recurrence

Variables	No recurrence	Recurrence	Test statistic	*P*
BMI (kg/m^2^**)**			χ^2^ = 8.29	0.028
18.5–24.99	80 (59.26)	5 (29.41)		
< 18.5	9 (6.67)	0		
25–29.99	39 (28.89)	11 (64.71)		
≥ 30	7 (5.18)	1 (5.88)		
Side			χ^2^ = 3.234	0.345
Right	53 (39.2)	6 (35.3)		
Left	73 (54.1)	8 (47.1)		
Bilateral	9 (6.7)	3 (17.6)		
Mass size at onset			χ^2^ = 1.031	0.797
< 1 cm	5 (3.7)	1 (5.9)		
1–2.9 cm	31 (22.9)	4 (23.5)		
3–5 cm	65 (48.2)	7 (41.2)		
> 5 cm	34 (25.2)	5 (29.4)		
Laboratory tests before treatment				
*C. kroppenstedtii*			χ^2^ = 1.317	0.453
Positive	35 (24.6)	1 (10)		
Negative	107 (75.4)	9 (90)		
White blood cell (WBC)				
mean, *10^9^/L	9.7 ± 3.4	10.1 ± 3.1	F = 0.197	0.658
C-reactive protein (CRP) mean, mg/L	12.3 ± 19.9	8.8 ± 6.9		
	F = 0.53	0.468		
Prolactin (PRL) mean, ng/mL	22.1 ± 19.8	27.2 ± 19.0	F = 0.924	0.338
Types of GM			χ^2^ = 4.857	0.2
Mass type	65 (48.2)	9 (52.9)		
Abscess type	59 (43.7)	7 (41.2)		
Refractory type	11 (8.1)	1 (5.9)		
Treatments			χ^2^ = 7.429	0.02
Medical + Breast lesion excision by minimally invasive surgery	17 (12.7)	2 (11.8)		
Medical + Breast lesion excision by open surgery	50 (37.0)	12 (70.6)		
Medication	67 (49.6)	3 (17.6)		
Observation	1 (0.7)	0		
Medication			χ^2^ = 7.175	0.018
Single steroids	77 (57.0)	7 (41.2)		
Single tubercle bacillus drug	20 (14.8)	0		
Combined medication	37 (27.5)	10 (58.8)		
Without medication	1 (0.7)	0		
Medication adherence			χ^2^ = 5.932	0.046
Low	17 (12.7)	6 (35.3)		
Median	63 (47.0)	8 (47.1)		
High	54 (40.3)	3 (17.6)		
Follow-up time, m	24.55	24.41	F = 0.015	0.901

**Table 6:** Risk factors for GM recurrence by multivariate analysis

Variables	Rate ratio	95% CI	*P*
*BMI (kg/m*^*2*^*)*			
< 24.99	1	Reference	0.166
25–30	3.467	0.949–12.666	0.060
≥ 30	3.245	0.269–39.211	0.355
*Treatments*			
Medication		Reference	
Medical + surgery	4.128	1.026–16.610	0.046
*Medication*			
Single medication		Reference	
Multiple medication	2.556	0.821–7.957	0.105
*Medication adherence*			
Low		Reference	0.046
Median	0.656	0.160–1.182	0.064
High	0.428	0.224–0.867	0.015

## Discussion and conclusion

This is the first study to report a case management model applied for GM patients. Although GM is a benign disease, its recurrence, one of the main challenges in the management of patients with the disease, has been reported to occur in 5%-50% of patients [10–12]. In our study, the recurrence rate of 11.2% is low in this range. Seventeen patients experienced recurrence, including ten with new lesions in the ipsilateral breast and seven with new lesions in the contralateral breast.

In recent years, the prevalence of granulomatous mastitis has been rapidly increasing, and the most affected patients are women of childbearing age [39]. In two studies, Freeman et al. reported that up to 86% of GM patients had a history of pregnancy in the past 5 years [38]. Prasad et al. reported that 73 patients with GM had a mean age of approximately 33 years and a history of childbirth 4.6 years before mastitis on average [40]. In our study, which had similar characteristics to previously reported studies, the median age of the patients was 32 years (range 22–48), 119 patients had a history of childbirth within the last 5 years, 15 patients had concurrent pregnancy, and 4 patients were currently breastfeeding. These findings indicated that hormones play an important role and may be related to the secretion theory, which has an important place in the pathophysiology of GM [12]. It has been postulated that GM results from a localized autoimmune response to the retained or extra vacated fat- or protein-rich secretions in the breast ducts in women of childbearing age due to previous hyperprolactinemia [41]. Therefore, the breast care for women of childbearing age deserves our attention.

**Fig. 2:**
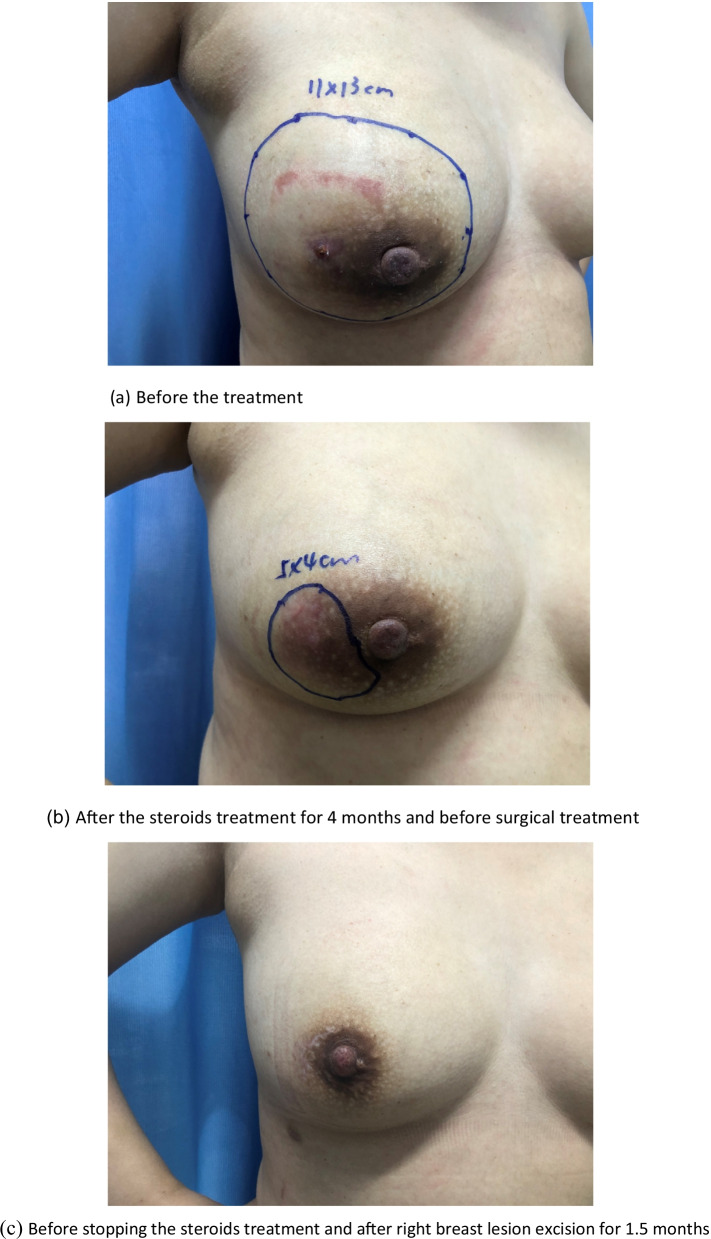
The effect of medical and surgical treatment in the case management. The underlined part of the figure shows the scope of the lesion located by ultrasound. **a** Before the treatment. **b** After the steroids treatment for 4 months and before surgical treatment. **c** Before stopping the steroids treatment and after right breast lesion excision for 1.5 months

**Fig. 3:**
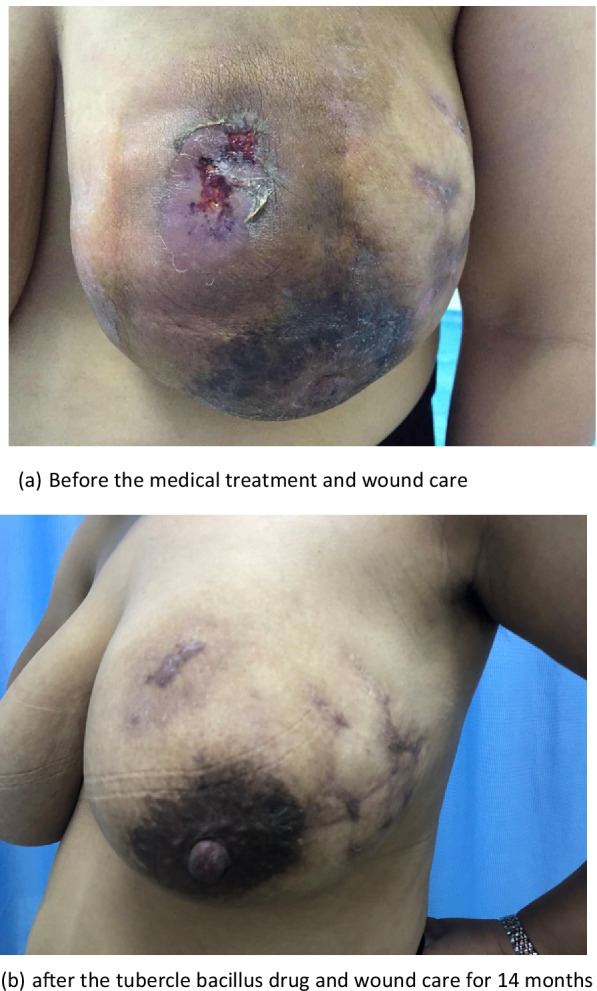
The effect of medical treatment in the case management. **a** Before the medical treatment and wound care. **b** After the tubercle bacillus drug and wound care for 14 months

GM patients mostly have mass and pain symptoms, and skin lesions and abscesses can be observed in mass localization. Findings such as fistula, erythema nodosum, and nipple or skin retraction can also be observed [1, 2, 35]. In many studies, the most common reported complaint at the time of the initial visit was a unilateral painful breast mass [35, 42]. Similarly, 98.7% of the patients had mass and pain complaints, and 92.1% of the patients presented with a unilaterally affected breast. The case managers made initial judgments and provided tentative guidance based on clinical presentations. At the initial visit, there were mass (74, 48.7%), abscess (66, 43.4%), and refractory types (12, 7.9%), which were not associated with recurrence in the later stages (*P* = 0.2). As the disease progressed, 10 mass type cases were actually abscess type cases, and 4 abscess type cases were actually refractory type cases. An important consideration for case managers is the care of the affected breast (shown in Fig. 2 and Fig. 3). Wound care should consist of managing drainage from fistulae with gauze and other nonadherent dressings. Tape should be avoided due to further abrasion and irritation of the skin [43]. Meanwhile, if a patient has a superficial abscess, a case manager should percutaneously perform puncture aspiration, and determine how deep the abscess is, while a mammographer, assisted by ultrasound guidance, performs puncture drainage, to create a path for the drainage of secretions and reduction of pressure in the inflamed area due to the accumulation of inflammatory fluid.

Comparing the most recent publications on GM to older studies, there is no new information on this benign breast disease. Therefore, the best management of this disease is still unclear [11, 12]. The usual treatment for GM is close observation, medical treatment, surgical management, or a combination of medication and surgery [3, 15, 44]. In the present study, only 1 (0.7%) patient recovered under observation, 82 (53.9%) recovered with medication and surgery (as shown in Fig. 2), and 69 (45.4%) recovered with medication alone (as shown in Fig. 3). Multivariate analysis revealed that medication and surgery was significantly associated with recurrence (RR = 4.128, 95% CI [1.026–16.610], *P* = 0.0046) (Table 6). Regarding the cause of recurrence, previous studies have ascribed the incompleteness of excision to the failure of surgical treatment, or inconsistent follow-up times. In this study, case managers assessed changes in the size of the breast mass and the proportion of the mass to the breast size and considered whether the patients could undergo surgical excision with minimal impact on the aesthetics of the breast. Breast lesion excision by minimally invasive surgery or open surgery was applied, which may have a risk of incomplete surgical excision. Akcan et al. and Yabanoğlu et al. reported that complete excision of the breast lesion or wide excision with or without medication achieved low recurrence rates [38, 45]; however, it is possible to cause damage to the breast due to the excessive removal of tissues. Therefore, the biggest problem with surgical treatment is the contradiction between the surgical effect and the postoperative aesthetic effect. Whether the surgical procedure that is chosen which increases the recurrence rate of GM requires further investigation.

Our study demonstrated that medical treatment is the most prevalent treatment, regardless of whether it is coupled with surgical treatment. Drug therapies have numerous side effects, such as Cushion's syndrome, skin rash, abnormal liver enzymes and abnormal uric acid and [46]. In our study, 8 (5.3%) patients suffered from skin rash, 11 (7.2%) had abnormal liver enzymes, 3 (2.0%) had abnormal uric acid, and 1 (0.7%) had edema on the lips and face (as shown in Table 3). In this stage, case managers served as a treatment team by linking physicians, pharmacists, dermatologists, obstetricians, and general practitioners. They immediately communicated with the multidisciplinary team, and then guided patients regarding their medications, and finally, most of the side effects disappeared within 1 week.

To the best of our knowledge, there are no studies investigating medication adherence in GM patients. In our study, it shown that the MMAS-8 was completed by 154 patients, with 39% who had high adherence, 46.4% who had medium adherence, and 14.6% who had low adherence. As a result of case manager guidance, the “low” medication adherence rate of GM patients was much lower than that of 30% and 50% of reported for adults with chronic disease [47, 48]. Furthermore, “high” medication adherence (RR = 0.428, 95% CI 0.224–0.867, *P* = 0.015) at 2 months after initial medication use was significantly associated with a lower rate of recurrence in multivariate analysis. At the initial stage, the case managers paid more attention to the changes in the patients’ breast symptoms than to patient medication adherence, and the guidance and supervision of medical staff to patient medication need improvement. Currently, several reports have demonstrated the importance of regular visits to a physician, adequate patient contact time in clinical practice, and patient education to improve medication adherence to treatment [49, 50].

Recent evidence indicates that the occurrence and recurrence of GM is associated with the *Corynebacterium* species, especially *Corynebacterium kroppenstedtii* [39, 51]. In our study, samples of *C. kroppenstedtii* were obtained by ultrasound guidance for the puncture or biopsy of breast abscesses or hypoechoic masses. Breast pus or tissues were used for bacterial culture, and the positive rate of *C. kroppenstedti* was only 23.69% (36/152). In different studies, the positive rate of *C. kroppenstedtii* varies considerably, mainly due to the detection techniques. Li et al. [52] reported that nanopore sequencing showed accurate *C. kroppenstedti* detection over the culture method in GM patients. Therefore, the need to improve detection techniques for the *Corynebacterium* species will facilitate the study of the relationship between GM and bacteria.

In this study, the results showed that 22 (14.47%) patients had excitant food before the onset of GM. The recent literature reports that bacterial interactions have been confirmed between the breast and gut [53, 54]. Li et al. hypothesized that imbalances among the external environment, host, and microbiota lead to the occurrence of GM as follows: External factors disturb the balance between the immune microenvironment and breast flora and induce the release of inflammatory factors and milk secretion, resulting in damage to the mammary epithelium. The positive feedback between the immune and inflammatory reactions eventually induces GM [13]. The consumption of stimulating foods may disrupt the intestinal flora and induce inflammation. Therefore, patients with GM should be given information regarding disease considerations related to diet, sleep, behaviors, etc., as shown in Table 1.

Our study has several limitations. First, it cannot be confirmed whether interesting factors such as dietary and lifestyle habits are related to the occurrence and recurrence of GM. Second, the effects of this case management model cannot be assessed by this study. Therefore, there are several directions for our next work, including developing targeted strategies based on the case management model and exploring the effectiveness of this model in GM patients.

In conclusion, this study identified some factors associated with the recurrence of the disease under a case management model. “Low” medication adherence was a significant risk factor for the recurrence of granulomatous mastitis. The patients treated with medication and surgery did not have a reduced recurrence rate compared to those treated with medication alone.

## Data Availability

The datasets generated and/or analyzed during the current study are not publicly available due to restrictions related to confidentiality i.e., they contain information that could compromise the privacy of research participants, but are available from the corresponding author on reasonable request.
